# The Notch-Regulated Ankyrin Repeat Protein Is Required for Proper Anterior–Posterior Somite Patterning in Mice

**DOI:** 10.1002/dvg.20813

**Published:** 2011-10-13

**Authors:** Luke T Krebs, Cara K Bradley, Christine R Norton, Jingxia Xu, Kathleen F Oram, Christa Starling, Michael L Deftos, Michael J Bevan, Thomas Gridley

**Affiliations:** 1 Center for Molecular Medicine, Maine Medical Center Research InstituteScarborough, Maine; 2 The Jackson LaboratoryBar Harbor, Maine; 3 Department of Immunology and Howard Hughes Medical Institute, University of WashingtonSeattle, Washington

**Keywords:** Notch pathway, negative feedback, rostrocaudal somite patterning

## Abstract

The Notch-regulated ankyrin repeat protein (Nrarp) is a component of a negative feedback system that attenuates Notch pathway-mediated signaling. In vertebrates, the timing and spacing of formation of the mesodermal somites are controlled by a molecular oscillator termed the segmentation clock. Somites are also patterned along the rostral-caudal axis of the embryo. Here, we demonstrate that *Nrarp*-deficient embryos and mice exhibit genetic background-dependent defects of the axial skeleton. While progression of the segmentation clock occurred in *Nrarp*-deficient embryos, they exhibited altered rostrocaudal patterning of the somites. In *Nrarp* mutant embryos, the posterior somite compartment was expanded. These studies confirm an anticipated, but previously undocumented role for the *Nrarp* gene in vertebrate somite patterning and provide an example of the strong influence that genetic background plays on the phenotypes exhibited by mutant mice. genesis 50:366–374, 2012. © 2011 Wiley Periodicals, Inc.

## INTRODUCTION

The Notch signaling pathway is an evolutionarily conserved intercellular signaling mechanism. Genes of the Notch family encode large transmembrane receptors that interact with membrane-bound ligands encoded by Delta/Serrate/Jagged family genes. The receptor/ligand interaction induces proteolytic cleavages that free the intracellular domain of the Notch receptor from the cell membrane. The Notch intracellular domain translocates to the cell nucleus, where it forms a complex with the recombination binding protein J (RBPJ) protein and other components of a transcription activation complex, leading to the expression of Notch target genes. One of these targets is the *Nrarp* (Notch-regulated ankyrin repeat protein) gene that encodes a 114 amino acid protein containing two ankyrin repeat motifs. Highly conserved Nrarp family genes have been described in mouse (Krebs *et al*.,^2001^), Xenopus (Lahaye *et al*.,^2002^; Lamar *et al*.,^2001^), zebrafish (Topczewska *et al*.,^2003^), and chicken (Wright *et al*.,^2009^). In addition to being a Notch target gene, whose expression is induced by Notch signal reception (Krebs *et al*.,^2001^; Lahaye *et al*.,^2002^; Lamar *et al*.,^2001^), the NRARP protein also negatively regulates Notch signaling, indicating that it is a component of a negative feedback loop that attenuates the Notch signal (Lahaye *et al*.,^2002^; Lamar *et al*.,^2001^; Yun and Bevan,^2003^).

In vertebrate embryos, timing and spacing of somite formation are controlled by a molecular oscillator termed the segmentation clock (Dequeant and Pourquie,^2008^; Gibb *et al*.,^2010^; Lewis *et al*.,^2009^), and *Nrarp* gene expression exhibits an oscillating pattern in presomitic mesoderm of mouse, chick, and zebrafish embryos (Dequeant *et al*.,^2006^; Sewell *et al*.,^2009^; Shifley *et al*.,^2008^; Wright *et al*.,^2009^). However, analysis of a *Nrarp* null mutant mouse reported vascular patterning defects in the retina (Phng *et al*.,^2009^) and normal progression of the segmentation clock (Wright *et al*.,^2009^). We describe here our analysis of mice with an independently generated null mutation of the *Nrarp* gene that display phenotypes not reported previously. We show that *Nrarp*^−/−^ mice exhibit genetic background-dependent defects in patterning of the axial skeleton that are due to expansion of the caudal compartment of the somite. These studies confirm the anticipated role of the *Nrarp* gene in vertebrate somite patterning and demonstrate that the failure to observe such a role in previous studies likely was due to differences in genetic background.

## RESULTS

### Disruption of the Mouse *Nrarp* Gene

The mouse *Nrarp* gene is comprised of a single exon (Pirot *et al*.,^2004^). The *Nrarp*^*tm1Grid*^ targeting vector deleted the entire coding sequence of the *Nrarp* gene, thus creating a *Nrarp* null allele (Supporting Information Fig. S1a). Mice homozygous for the *Nrarp*^*tm1Grid*^ mutant allele (henceforth referred to as *Nrarp*^−/−^ mice) were viable and fertile. *Nrarp*^−/−^ mice were recovered at expected Mendelian frequencies on both a mixed C57BL/6J X 129S1/SvImJ (B6/129) and a 129S1/SvImJ (129) genetic background (Table 1). Polymerase chain reaction (PCR) analysis demonstrated that the entire coding sequence of the *Nrarp* gene was deleted in *Nrarp*^−/−^ mice (Supporting Information Fig. S1c), confirming that the *Nrarp*^*tm1Grid*^ allele is a null allele.

**Table 1:** Viability at Wean of Nrarp^−/−^ and Control Littermate Mice

Genetic background	Nrarp^+/+^	Nrarp^+/−^	Nrarp^−/−^
B6/129 (*n* = 137)	35 (26%)	72 (52%)	30 (22%)
129 (*n* = 112)	35 (31%)	54 (48%)	23 (21%)

Abbreviations: B6/129, mixed C57BL/6J X 129S1/SvImJ; 129: 129S1/SvImJ.

### Axial Skeletal Defects in *Nrarp*^−/−^ Mice Are Dependent on Genetic Background

**FIG. 1:**
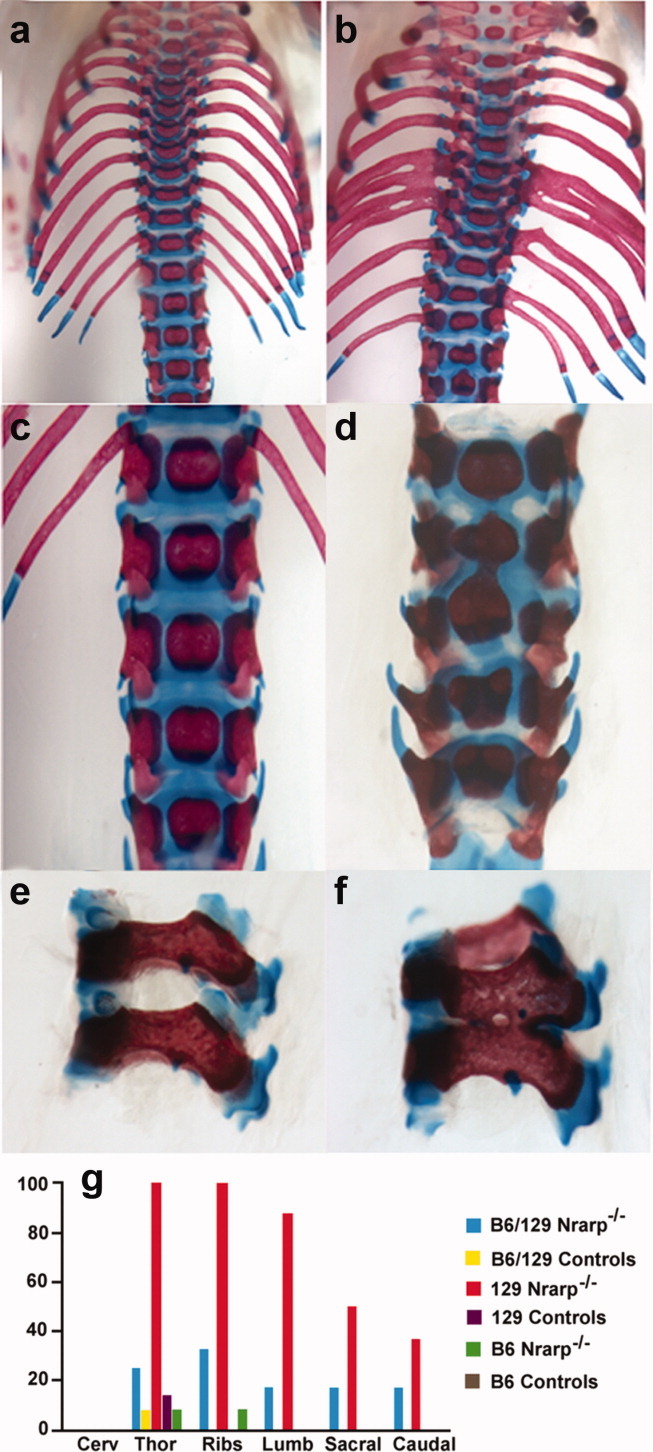
*Nrarp*^−/−^ mice exhibit axial skeletal defects. (**a–f**) Compared with control mice (a, c, e) at P0, skeletons of neonatal *Nrarp*^−/−^ mice (b, d, f) on the 129S1/SvImJ genetic background exhibit proximal rib fusions (b), fused vertebral bodies (d), and fused pedicles of the vertebrae (f). (**g**) Distribution and frequency of axial skeletal defects. The percentage of observed malformations along the vertebral column is displayed for the indicated genotypes and genetic backgrounds.

Analysis of *Nrarp* RNA expression has suggested a possible role for the *Nrarp* gene during somite formation and/or patterning in mice, and *Nrarp* RNA expression is altered in several Notch pathway mouse mutants that exhibit somite defects (Dequeant *et al*.,^2006^; Krebs *et al*.,^2001^; Sewell *et al*.,^2009^). We prepared alcian-blue/alizarin-red stained skeletons from *Nrarp*^−/−^ and littermate control mice. We initially analyzed skeletal preparations of postnatal day 0 (P0) *Nrarp*^−/−^ mutant mice on a mixed B6/129 background (F2 generation). Although we observed no obvious abnormalities of the cervical vertebrae, we did see small numbers of fusions between adjacent vertebrae in the thoracic, lumbar, sacral, and caudal regions (Table 2; Fig. 1g). Proximal rib fusions were also observed at low penetrance.

**Table 2:** Distribution and Frequency of Axial Skeletal Defects

	Cervical	Thoracic	Rib	Lumbar	Sacral	Caudal
(C57Bl/6J X 129S1/SvImJ)F2						
−/−	0% (0/12)	25% (3/12)	33% (4/12)	17% (2/12)	17% (2/12)	17% (2/12)
littermates	0% (0/14)	7% (1/14)	0% (0/14)	0% (0/14)	0% (0/14)	0% (0/14)
129S1/SvImJ						
−/−	0% (0/8)	100% (8/8)	100% (8/8)	87% (7/8)	50% (4/8)	37% (3/8)
littermates	0% (0/7)	14% (1/7)	0% (0/7)	0% (0/7)	0% (0/7)	0% (0/7)
C57BL/6J						
−/−	0% (0/12)	8% (1/12)	8% (1/12)	0% (0/12)	0% (0/12)	0% (0/12)
littermates	0% (0/38)	0% (0/38)	0% (0/38)	0% (0/38)	0% (0/38)	0% (0/38)

To determine whether the skeletal defects observed in *Nrarp*^−/−^ mice could be influenced by genetic background, we examined skeletons from *Nrarp*^−/−^ mice on a 129S1/SvImJ background (Fig. 1). We observed an increase in the penetrance and expressivity of vertebral abnormalities in the thoracic, lumbar, sacral, and caudal regions on the 129 genetic backgrounds (Fig. 1g). Rib fusions (Fig. 1b) observed in *Nrarp*^−/−^ mice on the 129S1/SvImJ background were similar to, but more severe than, those observed on the mixed genetic background. On both mixed and 129S1/SvImJ backgrounds, we typically observed fusions of adjacent vertebral bodies (Fig. 1d) and pedicles (Fig. 1f).

As the observed skeletal abnormalities were less severe on the mixed B6/129 background than on a 129S1/SvImJ background, we examined the effects of *Nrarp* deficiency on a predominantly C57BL/6J genetic background. (C57BL/6J X 129S1/SvImJ)F1 *Nrarp* mutant heterozygotes were backcrossed to C57BL/6J mice to the N4 generation and were then intercrossed to generate *Nrarp*^−/−^ mutants (approximately 94% C57BL/6J background). The number and frequency of skeletal abnormalities observed on the C57BL/6J background was dramatically decreased (Fig. 1g). No skeletal abnormalities were seen in the lumbar, sacral, or caudal regions. In *Nrarp*^−/−^ mice on the C57BL/6J background, the only observed defects were on the ninth and tenth thoracic vertebrae and ribs (Table 2).

### *Nrarp*^−/−^ Embryos Show Defects in Anterior–Posterior Somite Patterning, But Exhibit Progression of the Segmentation Clock

**FIG. 2:**
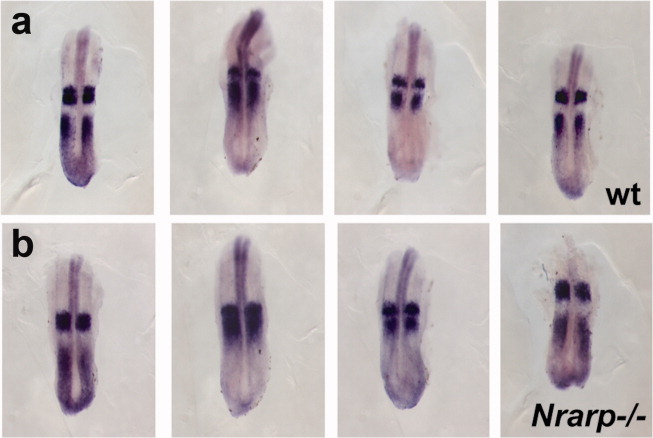
Segmentation clock progression occurs in *Nrarp*^−/−^ embryos. *Lfng* expression in the presomitic mesoderm was assessed by whole mount in situ hybridization of wild type (**a**) and *Nrarp*^−/−^ (**b**) embryos at E9.5. Several different patterns of cycling *Lfng* expression are shown.

In vertebrates, the timing and spacing of somite formation is controlled by a molecular oscillator termed the segmentation clock (Dequeant and Pourquie,^2008^; Gibb *et al*.,^2010^; Lewis *et al*.,^2009^). The *Nrarp* gene exhibits an oscillating pattern of expression in the presomitic mesoderm of mouse, chick, and zebrafish embryos (Dequeant *et al*.,^2006^; Sewell *et al*.,^2009^; Shifley *et al*.,^2008^; Wright *et al*.,^2009^), suggesting the possibility that Nrarp function might be required for progression of the segmentation clock or for other aspects of somite patterning. To determine whether the progression of the segmentation clock occurred in *Nrarp*^−/−^ embryos on the 129S1/SvImJ background, we examined *Lfng* RNA expression by whole mount in situ hybridization (Fig. 2). Although we cannot exclude that there may be small differences in the oscillating expression patterns of *Lfng* expression between *Nrarp*^−/−^ and control littermate embryos, this analysis demonstrated progression of the somite clock in *Nrarp*^−/−^ embryos, indicating that *Nrarp* function was not absolutely required for cyclic gene expression. A similar finding has been made in chick embryos electroporated with either gain or loss of function *cNrarp* expression constructs, and in a previously generated *Nrarp* null mutant mouse (Wright *et al*.,^2009^).

**FIG. 3:**
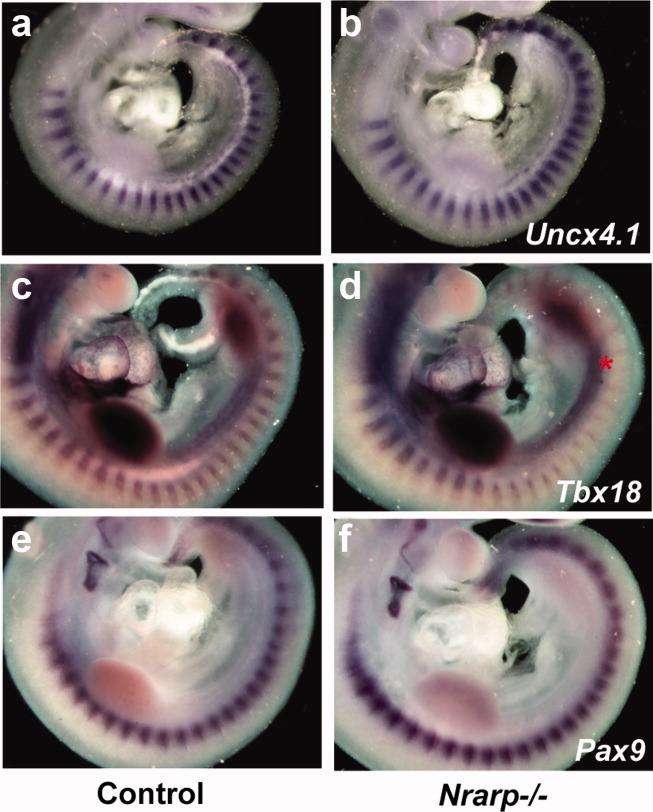
Anterior–posterior somite patterning defects in *Nrarp*^−/−^ embryos. (**a**, **b**) *Uncx4.1* expression in the posterior somite compartment was expanded in *Nrarp*^−/−^ embryos (b) at E9.5. (**c**,**d**) *Tbx18* expression in the anterior somite compartment was downregulated (red asterisk) in *Nrarp*^−/−^ embryos (d). (**e**, **f**) Hybridization with a *Pax9* riboprobe, a sclerotome marker expressed at higher levels in the posterior somite compartment. *Nrarp*^−/−^ embryos (f) exhibit an expansion of this posterior domain of high-level *Pax9* expression.

**FIG. 4:**
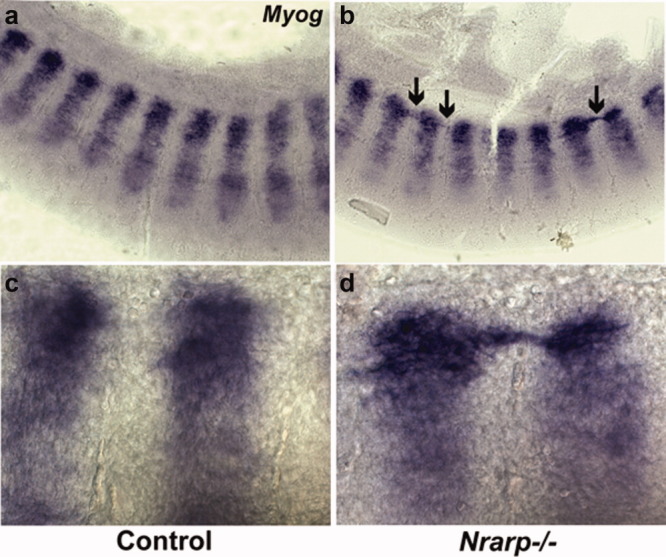
Myotome defects in *Nrarp*^−/−^ embryos. (**a**–**d**) Dissected and flat-mounted paraxial mesoderm from E10.5 control and *Nrarp*^−/−^ embryos hybridized with a myogenin (*Myog*) riboprobe, a myotome marker. *Nrarp*^−/−^ embryos exhibit expansion and fusion of myotomes (arrows in b).

**FIG. 5:**
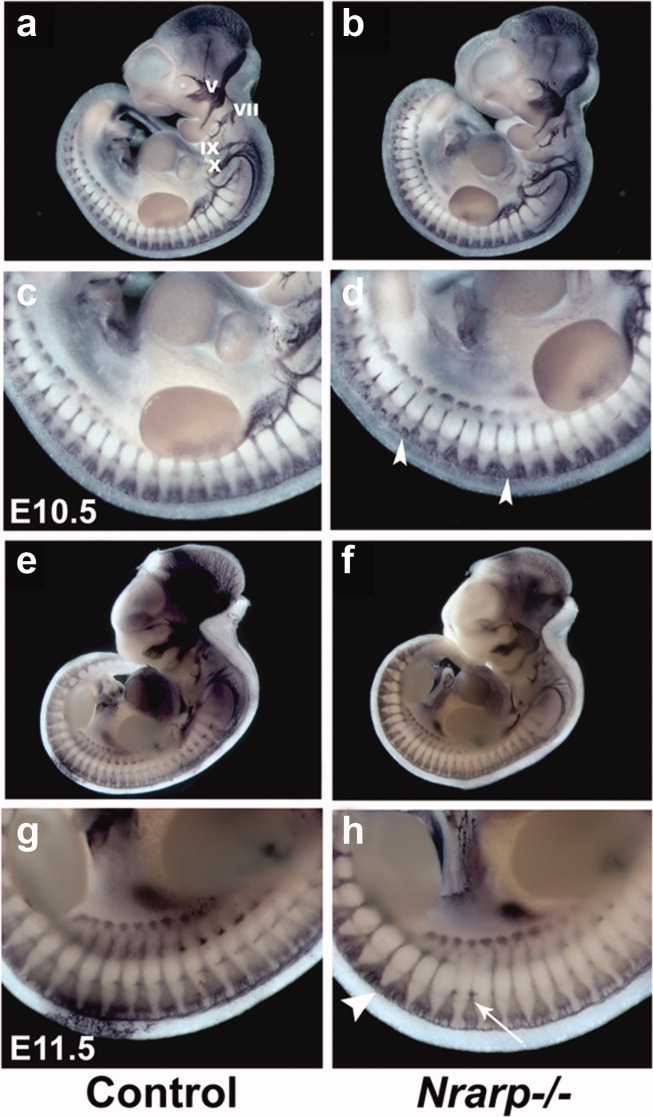
Dorsal root ganglia and spinal nerve defects in *Nrarp*^−/−^ embryos. (**a**–**h**) Whole mount immunohistochemistry with anti-165 kDa neurofilament antibody of E10.5 (a–d) and E11.5 (e–h) embryos. *Nrarp*^−/−^ embryos exhibit dorsal root ganglia fusions (arrowheads in d and h) and defects in projection of the spinal nerves (arrow in h), which normally traverse only the rostral somite compartment. The V, VII, IX, and X cranial ganglia are marked in (a).

As segmentation clock progression occurred in the *Nrarp*^−/−^ mutants, we next assessed whether *Nrarp*^−/−^ embryos exhibited defects in rostrocaudal somite patterning by analyzing expression of several genes exhibiting specific patterns of expression in the somite. The *Uncx4.1* gene encodes a paired-related homeobox protein that is expressed in the posterior compartment of the somite (Mansouri *et al*.,^1997^), whereas the *Tbx18* gene encodes a T-box protein expressed in the anterior compartment (Kraus *et al*.,^2001^). In E10.5 *Nrarp*^−/−^ embryos, the *Uncx4.1* expression domain was expanded anteriorly (Fig. 3b; also see Supporting Information Fig. S2b), while *Tbx18* expression was reduced (Fig. 3d; Supporting Information Fig. S2d). Expansion of the posterior somite compartment was also indicated by the expression of the sclerotome marker *Pax9* (Fig. 3f; Supporting Information Fig. S2f) and the myotome marker myogenin (*Myog*; Fig. 4). Analysis of expression of the 165-kDa neurofilament protein revealed fusions of dorsal root ganglia and defects in projection of the spinal nerves (Fig. 5), which normally traverse only the rostral somite compartment. These defects in the peripheral nervous system are consistent with expansion of the posterior somite compartment in *Nrarp*^−/−^ embryos.

### *Nrarp*^−/−^ Embryos Exhibit Increased Expression of Cleaved NOTCH1 Protein

**FIG. 6:**
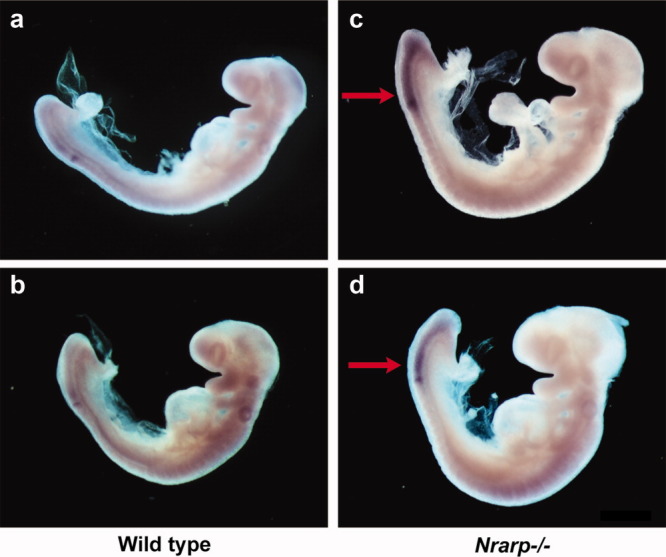
Expression of cleaved-Notch1 protein is increased in presomitic mesoderm and newly formed somites of *Nrarp*^−/−^ embryos. (**a–d**) Whole mount immunohistochemistry with anti-Val1744 antibody of E9.5 wild type littermate (a, b) and *Nrarp*^−/−^ (c, d) embryos. Expression of the cleaved form of the NOTCH1 protein (recognized by the anti-Val1744 antibody) is increased in the presomitic mesoderm and newly formed somites of *Nrarp*^−/−^ embryos (red arrows).

Previous studies have demonstrated that the NRARP protein negatively regulates Notch signaling by binding and destabilizing the cleaved, activated intracellular domain of the NOTCH1 protein (Ishitani et al.,^2005^; Lamar et al.,^2001^). We assessed expression of the cleaved form of the NOTCH1 protein by whole mount immunohistochemistry in *Nrarp*^−/−^ and wild type littermate control embryos. *Nrarp*^−/−^ embryos exhibited increased cleaved NOTCH1 protein expression in presomitic mesoderm and somites (Fig. 6), supporting the model that the NRARP protein functions as part of a negative feedback loop regulating the duration of the Notch signal in the paraxial mesoderm.

### *Nrarp*^−/−^Mice Exhibit Growth Retardation, But Not Obvious Hematopoietic or Craniofacial Defects

**FIG. 7:**
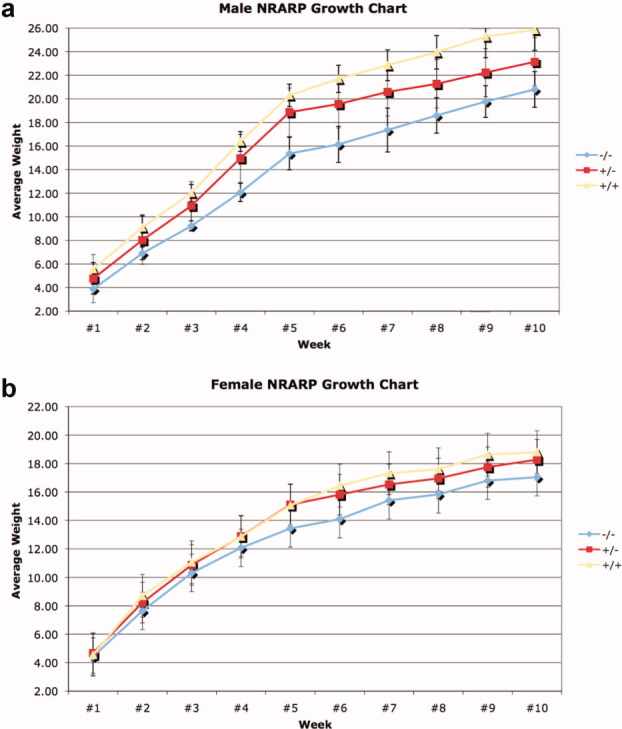
Growth curves of *Nrarp*^−/−^ and control littermate mice. Postnatal growth charts of male (**a**) and female (**b**) *Nrarp*^−/−^ and littermate control mice.

We also analyzed *Nrarp*^−/−^ mice for other phenotypes that have been suggested by either gain of function studies in mice or by analysis of *Nrarp*-family genes in other vertebrates. These studies revealed that *Nrarp*^−/−^ mice exhibit modest postnatal growth retardation (Fig. 7). Previous gain of function experiments in mice have shown that constitutive *Nrarp* expression in hematopoietic stem cells resulted in a block in T cell lineage commitment and progression through the early stages of thymocyte maturation (Yun and Bevan,^2003^). To determine whether *Nrarp* loss of function led to any obvious hematopoietic defects, we analyzed differentiation of the major hematopoietic lineages in *Nrarp*^−/−^ mice. We assessed hematopoietic stem cells, early T cells (DN1-DN4), T cells (CD4/CD8), B cells, myeloid cells, and erythroid cells. No obvious differences in hematopoietic development within the thymus, spleen, and bone marrow were observed in *Nrarp*^+/+^, *Nrarp*^+/−^, and *Nrarp*^−/−^ mice (*n* = 4 for each genotype) on the 129S1/SvImJ background (Table 3). Zebrafish *nrarp-a* morphants exhibit defective formation of cranial cartilage, which is derived from the cranial neural crest (Ishitani *et al*.,^2005^). Examination of skulls from *Nrarp*^−/−^ neonatal mice revealed no obvious defects in craniofacial development or morphogenesis at P0 (Supporting Information Fig. S3).

**3:** Hematopoietic Differentiation in Nrarp^−/−^ and Control Littermate Mice

Tissue	Cell type	Stain	Nrarp^+/+^	Nrarp^+/−^	Nrarp^−/−^
Thymus	Early T (DN1)	CD25−/CD44+/Lin−	26.5 ± 4.6	31.4 ± 4.9	27.1 ± 3.2
	Early T (DN2)	CD25+/CD44+/Lin−	8.8 ± 0.9	8.9 ± 0.5	9.3 ± 1.4
	Early T (DN3)	CD25+/CD44−/Lin−	35.9 ± 5.8	34.9 ± 7.0	38.1 ± 5.2
	Early T (DN4)	CD25−/CD44−/Lin−	28.8 ± 8.0	24.9± 3.8	25.5 ± 6.3
	T (CD4)	CD4+/CD8−	8.4 ± 1.0	8.5 ± 0.9	9.9 ± 1.2
	T (DP)	CD4+/CD8+	87.2 ± 1.5	87.0 ± 0.9	85.0± 1.5
	T (CD8)	CD4−/CD8+	2.5 ± 0.3	2.4 ± 0.2	2.7 ± 0.1
	T (DN)	CD4−/CD8−	1.9 ± 0.5	2.2 ± 0.4	2.4 ± 0.6
Spleen	T (CD4)	CD4+/CD8−	20.4 ± 2.4	23.5 ± 3.2	23.7 ± 1.8
	T (DP)	CD4+/CD8+	0.5 ± 0.1	0.8 ± 0.4	0.6 ± 0.1
	T (CD8)	CD4−/CD8+	9.0 ± 0.8	10.2 ± 1.6	9.9 ± 0.8
	T (DN)	CD4−/CD8−	70.1 ± 2.9	65.6 ± 5.0	66.0 ± 2.0
	Myeloid	Mac1+	6.3 ± 0.3	6.9 ± 2.2	6.3 ± 1.1
	B cells	B220+/CD19+	57.2 ± 3.2	57.8 ± 2.1	57.8 ± 3.1
Bone marrow	Myeloid	Mac1+	45.3 ± 4.4	44.2 ± 3.3	42.4 ± 5.7
	Erythroid	CD71+/Ter119+	26.2 ± 3.2	25.7 ± 2.2	26.0 ± 6.7
	B cells	B220+/CD19+	26.3 ± 2.9	24.8 ± 4.0	28.9 ± 4.9
	HSC	cKit+/Sca1+/Lin−	2.9 ± 0.2	2.9 ± 0.6	2.5 ± 0.2

Abbreviations: DN, double negative; DP, double positive. For myeloid cells, erythroid cells, B cells, and hematopoietic stem cells (HSC), data are presented as percentage ± standard deviation of cells with the indicated marker expression profile. For early T cells (DN1–DN4) and T cells (CD4, DP, CD8, DN), data are presented as the percentage ± standard deviation of total early T cells (DN cells) or T cells, respectively. Cells were isolated from four mice from each of the indicated genotypes.

## DISCUSSION

Our work demonstrates that the NRARP protein is required, in a genetic background dependent manner, for anterior–posterior somite patterning in mice. Notch signaling is active in the posterior (caudal) compartment of the somite (Oginuma et al.,^2008^; Takahashi et al.,^2003^; Takahashi et al.,^2010^), and *Nrarp*^−/−^ embryos exhibit expansion of the posterior somite compartment. A similar, albeit more severe, phenotype is displayed by mouse embryos with constitutive expression of the NOTCH1 intracellular domain (NICD) throughout the presomitic mesoderm (Feller *et al*.,^2008^). These embryos exhibit expression of *Uncx4.1* throughout the epithelial somite and loss of *Tbx18* expression. The phenotype exhibited by *Nrarp*^−/−^ embryos and mice is consistent with the model that the NRARP protein functions as a component of a negative feedback loop to destabilize NICD and downregulate the Notch signal, preventing expansion of the Notch signal into the anterior somite domain.

In addition, our studies provide another example of the strong influence that genetic background has on the phenotypes exhibited by mutant mouse embryos (e.g., Cozzi *et al*.,^2011^; Kiernan *et al*.,^2007^; Threadgill *et al*.,^1995^; Vervoort *et al*.,^2010^). A goal of future studies will be to determine which genes on the 129 genetic backgrounds sensitize *Nrarp*^−/−^ embryos to exhibit axial skeletal defects. These studies may have important medical implications, because mutations in a number of Notch pathway components or downstream targets cause the human axial skeletal disorder spondylocostal dysostosis (Dunwoodie,^2009^).

## MATERIALS AND METHODS

### Gene Targeting and Mutant Mice

The *Nrarp*^*tm1Grid*^ targeting vector was constructed from strain 129S6/Sv BAC clones, and deletes the entire coding sequence of the NRARP protein (from 93 base pairs 5′of the ATG start site to 1,286 bases 3′of the last base in the coding sequence of the NRARP protein). To generate the right arm of the *Nrarp*^*tm1Grid*^ targeting vector, a 3.1-kb BamH1-SacII genomic subclone was inserted into pBluescript II KS (Invitrogen) containing a diphtheria toxin expression cassette for negative selection against random integration of the targeting vector and a neomycin expression cassette for positive selection. A 5.2-kb KpnI-XhoI genomic subclone was inserted to generate the left arm of the targeting vector. The left arm XhoI site is located 93 bases 5′of the NRARP protein ATG start codon, while the BamHI site in the right arm is 1,286 bases 3′of the last base in the coding sequence of the NRARP protein.

The *Nrarp*^*tm1Grid*^ targeting construct was electroporated into R1 embryonic stem cells, and germ-line transmission was obtained for two independently targeted clones. The *Nrarp*^*tm1Grid*^ targeted allele retains the neomycin expression cassette. PCR primers for the wild type *Nrarp* allele were 5′TAGCTCTGCGGCAACATGA 3′ and 5′AGAGACTTAGCCCGATTTCC3′, yielding an amplification product of 458 base pairs (bp). These two wild type primers span the coding sequence of the single exon *Nrarp* gene. These primers cover from 339 bp to 797 bp of the *Nrarp* gene. The ATG start site for the NRARP protein is at 354 bp, and the stop codon is at 698 bp. PCR primers for the *Nrarp*^*tm1Grid*^ mutant allele were 5′TGCTGATCTCGTTCTTCAGG3 ′ and 5′TCGCCTTCTA TCGCCTTCTTG3′ (located within the neomycin expression cassette), yielding a product of 440 base pairs.

### In Situ Hybridization and Immunohistochemistry

Whole mount in situ hybridization (Krebs *et al*.,^2001^) and whole mount immunohistochemistry (Swiatek and Gridley,^1993^) with anti-165 kDa neurofilament antibody (monoclonal antibody 2H3, Developmental Studies Hybridoma Bank) were performed as described previously. For some embryos exposed to whole mount in situ hybridization, paraxial mesoderm was removed, bisected with tungsten needles, and flat mounted on glass slides for photography. Whole mount immunohistochemistry with the cleaved NOTCH1 (anti-Val1744) antibody (Cell Signaling Technology) was performed as described (Feller *et al*.,^2008^).

### Skeletal Preparations

Alizarin red/alcian blue-stained skeletal preparations were performed as described previously (Murray *et al*.,^2007^).

### Flow Cytometry

Flow cytometry analysis of hematopoietic lineages was conducted on the thymus, spleen, and bone marrow from strain 129S1/SvImJ background *Nrarp*^+/+^, *Nrarp*^+/−^, and *Nrarp*^−/−^ mice at 4–5 months of age (*n* = 4 for each genotype). Cell lineages analyzed and antibody markers used were hematopoietic stem cells (cKit, Sca1, Lin [NK1.1, CD3, CD4, CD8, CD19, Gr1, Mac1, Ter119]), early T cells (CD25, CD44, Lin), T cells (CD4, CD8, TCRβ), B cells (CD19, B220), myeloid cells (Gr1, Mac1), and erythroid cells (CD71, Ter119).

## Supplementary material

Supporting Information Fig. S1. Generation of a Nrarp null allele. (A) Schematic representation of the wild type Nrarp allele, the Nrarptm1Grid targeting vector, and the targeted Nrarptm1Grid allele. The single exon of the Nrarp gene is indicated by the gray box, with coding sequence designated by the black box. The Nrarptm1Grid targeting vector deletes all Nrarp coding sequences. LoxP sequences flanking the neomycin selection cassette are marked by triangles. A diphtheria toxin (DT) cassette was included for negative selection of randomly integrated clones. Hybridization probes used for Southern blot analysis are indicated. (B) Southern blot analysis of Nrarp intercross progeny. EcoRI-digested genomic DNA was hybridized with the 3' probe (as indicated in A), yielding 6.0 kb wild type and 4.5 kb mutant bands. Genotypes are indicated at top of lane. (C) Genomic PCR analysis of DNA from Nrarp intercross progeny. Genotypes are indicated at top of lane. Since the wild type PCR primers span the coding sequence of the Nrarp gene, these results demonstrate that the entire coding sequence of the Nrarp gene has been deleted in the Nrarptm1Grid allele. M, markers.

Supporting Information Fig. S2. Nrarp-/- embryos exhibit expansion of the posterior somite compartment. (A-F) Dissected and flat-mounted paraxial mesoderm from E10.5 control and Nrarp-/- embryos hybridized in whole mount with riboprobe markers for the anterior and posterior somitic compartments. (A,B) Nrarp-/- embryos exhibit expansion of the Uncx4.1 expression domain, which is a marker of the posterior somite compartment. (C,D) Nrarp-/- embryos exhibit a reduction of the Tbx18 expression domain, which is a marker of the anterior somite compartment. (E,F) Hybridization with a Pax9 riboprobe, a sclerotome marker expressed at higher levels in the posterior somite compartment. Nrarp-/- embryos exhibit an expansion of the domain of high-level Pax9 expression, indicating an expansion of the posterior somite compartment. Anterior is towards the left in all panels.

Supporting Information Fig. S3. Normal craniofacial development of Nrarp-/- mice. (A,B) Alizarin red/alcian blue-stained skulls of control and Nrarp-/- mice at P0. The Nrarp-/- neonatal mice (B) revealed no obvious defects in craniofacial development or morphogenesis.

## References

[b1] Cozzi E, Ackerman KG, Lundequist A, Drazen JM, Boyce JA, Beier DR (2011). The naive airway hyperresponsiveness of the A/J mouse is Kit-mediated. Proc Natl Acad Sci USA.

[b2] Dequeant ML, Glynn E, Gaudenz K, Wahl M, Chen J, Mushegian A, Pourquie O (2006). A complex oscillating network of signaling genes underlies the mouse segmentation clock. Science.

[b3] Dequeant ML, Pourquie O (2008). Segmental patterning of the vertebrate embryonic axis. Nat Rev Genet.

[b4] Dunwoodie SL (2009). The role of Notch in patterning the human vertebral column. Curr Opin Genet Dev.

[b5] Feller J, Schneider A, Schuster-Gossler K, Gossler A (2008). Noncyclic Notch activity in the presomitic mesoderm demonstrates uncoupling of somite compartmentalization and boundary formation. Genes Dev.

[b6] Gibb S, Maroto M, Dale JK (2010). The segmentation clock mechanism moves up a notch. Trends Cell Biol.

[b7] Ishitani T, Matsumoto K, Chitnis AB, Itoh M (2005). Nrarp functions to modulate neural-crest-cell differentiation by regulating LEF1 protein stability. Nat Cell Biol.

[b8] Kiernan AE, Li R, Hawes NL, Churchill GA, Gridley T (2007). Genetic background modifies inner ear and eye phenotypes of jag1 heterozygous mice. Genetics.

[b9] Kraus F, Haenig B, Kispert A (2001). Cloning and expression analysis of the mouse T-box gene Tbx18. Mech Dev.

[b10] Krebs LT, Deftos ML, Bevan MJ, Gridley T (2001). The *Nrarp* gene encodes an ankyrin-repeat protein that is transcriptionally regulated by the Notch signaling pathway. Dev Biol.

[b11] Lahaye K, Kricha S, Bellefroid E (2002). XNAP, a conserved ankyrin repeat-containing protein with a role in the Notch pathway during Xenopus primary neurogenesis. Mech. Dev.

[b12] Lamar E, Deblandre G, Wettstein D, Gawantka V, Pollet N, Niehrs C, Kintner C (2001). Nrarp is a novel intracellular component of the Notch signaling pathway. Genes Dev.

[b13] Lewis J, Hanisch A, Holder M (2009). Notch signaling, the segmentation clock, and the patterning of vertebrate somites. J Biol.

[b14] Mansouri A, Yokota Y, Wehr R, Copeland NG, Jenkins NA, Gruss P (1997). Paired-related murine homeobox gene expressed in the developing sclerotome, kidney, and nervous system. Dev Dyn.

[b15] Murray SA, Oram KF, Gridley T (2007). Multiple functions of Snail family genes during palate development in mice. Development.

[b16] Oginuma M, Niwa Y, Chapman DL, Saga Y (2008). Mesp2 and Tbx6 cooperatively create periodic patterns coupled with the clock machinery during mouse somitogenesis. Development.

[b17] Phng LK, Potente M, Leslie JD, Babbage J, Nyqvist D, Lobov I, Ondr JK, Rao S, Lang RA, Thurston G, Gerhardt H (2009). Nrarp coordinates endothelial Notch and Wnt signaling to control vessel density in angiogenesis. Dev Cell.

[b18] Pirot P, van Grunsven LA, Marine JC, Huylebroeck D, Bellefroid EJ (2004). Direct regulation of the Nrarp gene promoter by the Notch signaling pathway. Biochem Biophys Res Commun.

[b19] Sewell W, Sparrow DB, Smith AJ, Gonzalez DM, Rappaport EF, Dunwoodie SL, Kusumi K (2009). Cyclical expression of the Notch/Wnt regulator Nrarp requires modulation by Dll3 in somitogenesis. Dev Biol.

[b20] Shifley ET, Vanhorn KM, Perez-Balaguer A, Franklin JD, Weinstein M, Cole SE (2008). Oscillatory lunatic fringe activity is crucial for segmentation of the anterior but not posterior skeleton. Development.

[b21] Swiatek PJ, Gridley T (1993). Perinatal lethality and defects in hindbrain development in mice homozygous for a targeted mutation of the zinc finger gene Krox20. Genes Dev.

[b22] Takahashi J, Ohbayashi A, Oginuma M, Saito D, Mochizuki A, Saga Y, Takada S (2010). Analysis of Ripply1/2-deficient mouse embryos reveals a mechanism underlying the rostro-caudal patterning within a somite. Dev Biol.

[b23] Takahashi Y, Inoue T, Gossler A, Saga Y (2003). Feedback loops comprising Dll1, Dll3 and Mesp2, and differential involvement of Psen1 are essential for rostrocaudal patterning of somites. Development.

[b24] Threadgill DW, Dlugosz AA, Hansen LA, Tennenbaum T, Lichti U, Yee D, LaMantia C, Mourton T, Herrup K, Harris RC (1995). Targeted disruption of mouse EGF receptor: effect of genetic background on mutant phenotype. Science.

[b25] Topczewska JM, Topczewski J, Szostak A, Solnica-Krezel L, Hogan BL (2003). Developmentally regulated expression of two members of the Nrarp family in zebrafish. Gene Expr Patterns.

[b26] Vervoort R, Ceulemans H, Van Aerschot L, D'Hooge R, David G (2010). Genetic modification of the inner ear lateral semicircular canal phenotype of the Bmp4 haplo-insufficient mouse. Biochemical and biophysical research communications.

[b27] Wright D, Ferjentsik Z, Chong SW, Qiu X, Jiang YJ, Malapert P, Pourquie O, Van Hateren N, Wilson SA, Franco C, Gerhardt H, Dale JK, Maroto M (2009). Cyclic Nrarp mRNA expression is regulated by the somitic oscillator but Nrarp protein levels do not oscillate. Dev Dyn.

[b28] Yun TJ, Bevan MJ (2003). Notch-regulated ankyrin-repeat protein inhibits Notch1 signaling: multiple Notch1 signaling pathways involved in T cell development. J Immunol.

