# Supplementary figures and images for: The Notch-Regulated Ankyrin Repeat Protein Is Required for Proper Anterior–Posterior Somite Patterning in Mice
Source: Genesis. 2011 Oct 13;50(4):366–74. doi: 10.1002/dvg.20813 (PMC3314717; doi:10.1002/dvg.20813)

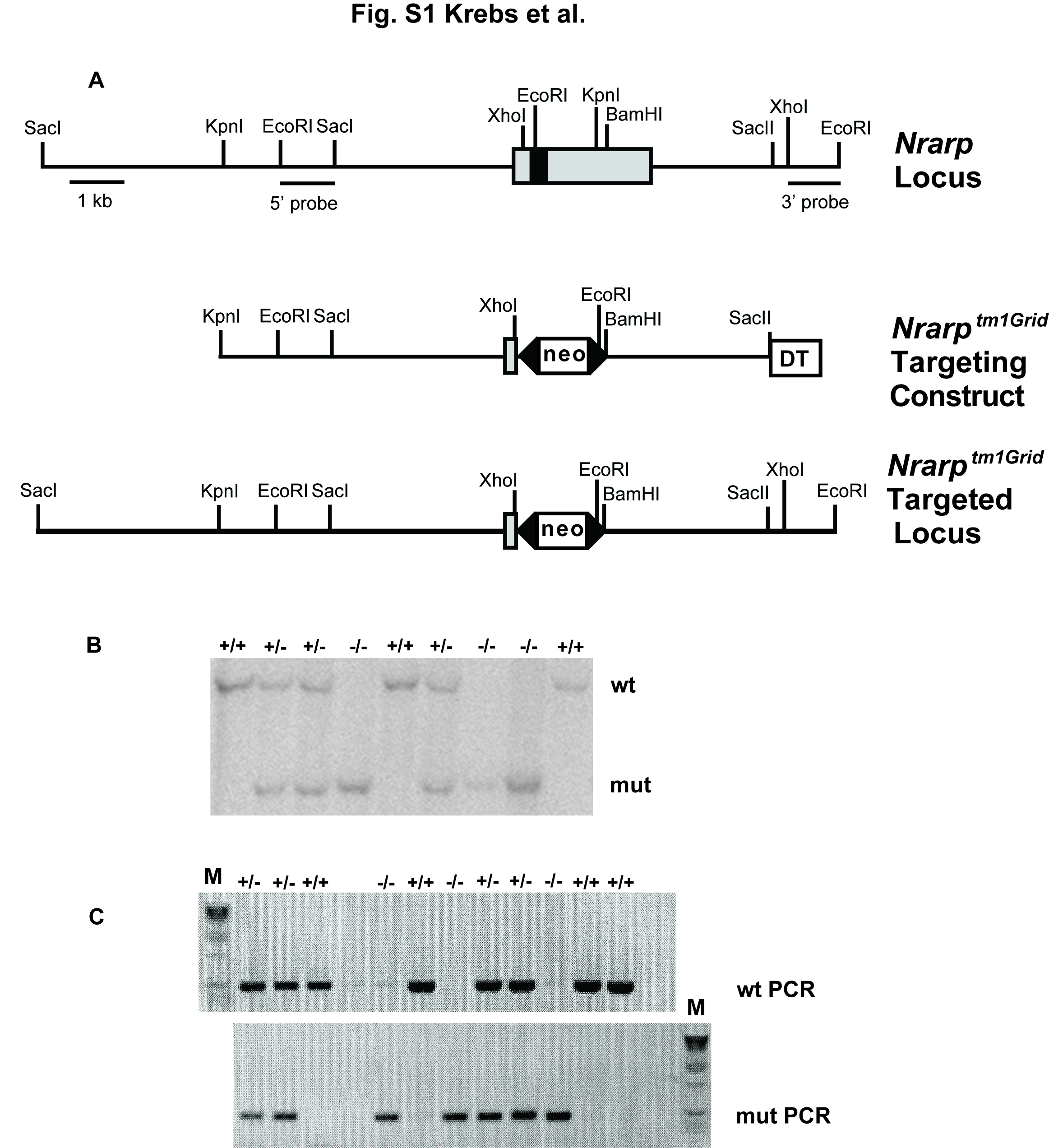

Supplement: Supplementary file 1 [file dvg0050-0366-SD1.tif]

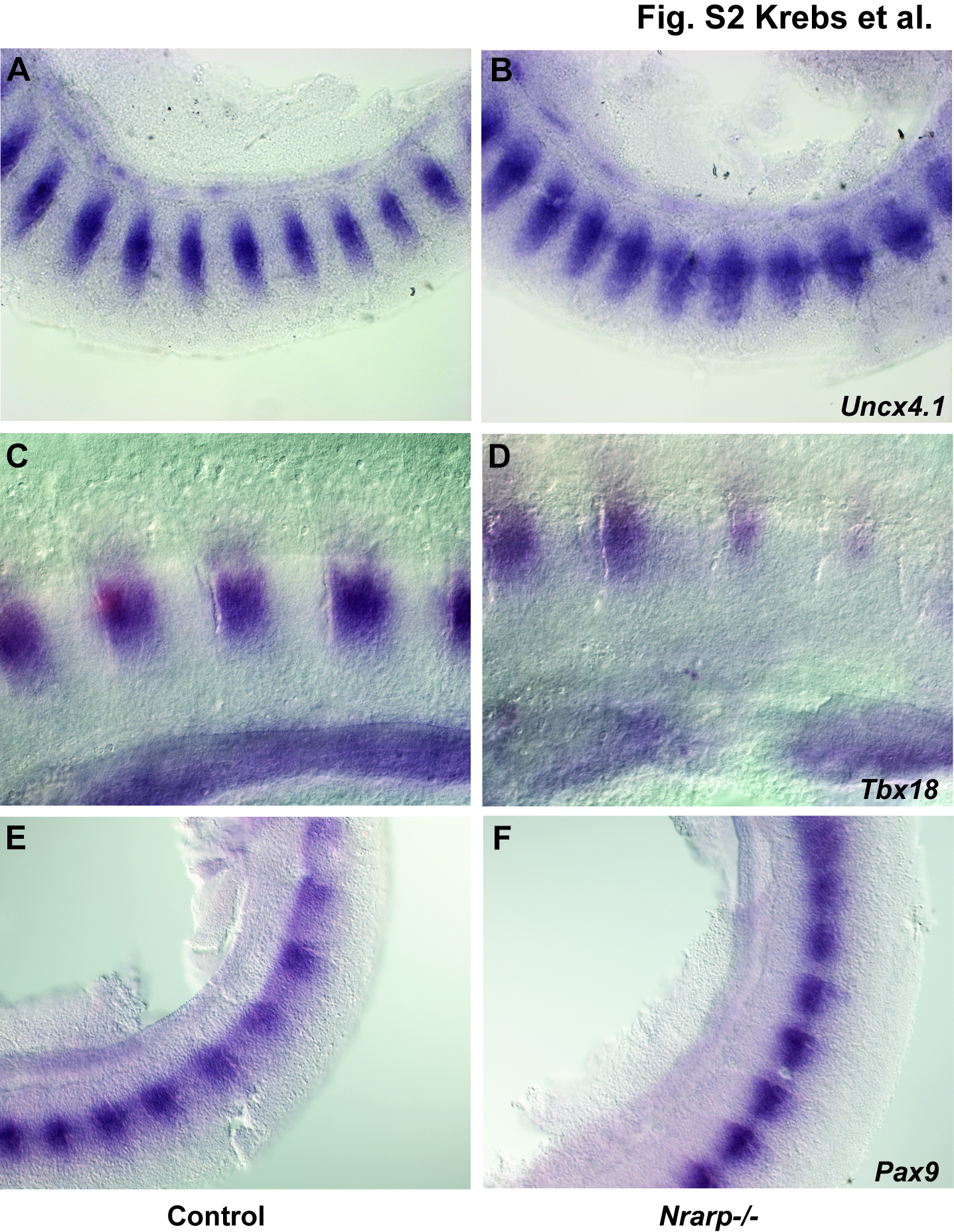

Supplement: Supplementary file 2 [file dvg0050-0366-SD2.tif]

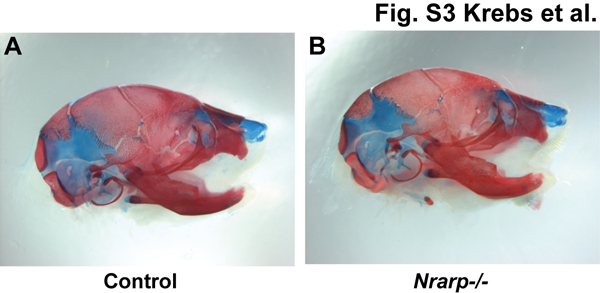

Supplement: Supplementary file 3 [file dvg0050-0366-SD3.tif]
